# Silencing or inhibition of endoplasmic reticulum aminopeptidase 1 (ERAP1) suppresses free heavy chain expression and Th17 responses in ankylosing spondylitis

**DOI:** 10.1136/annrheumdis-2014-206996

**Published:** 2015-06-30

**Authors:** Liye Chen, Anna Ridley, Ariane Hammitzsch, Mohammad Hussein Al-Mossawi, Helen Bunting, Dimitris Georgiadis, Antoni Chan, Simon Kollnberger, Paul Bowness

**Affiliations:** 1 Nuffield Department of Orthopaedics, Rheumatology and Musculoskeletal Sciences, University of Oxford, Oxford, UK; 2 Department of Chemistry, University of Athens, Athens, Greece; 3 Department of Rheumatology, Royal Berkshire Hospital, Reading, UK

**Keywords:** Ankylosing Spondylitis, Autoimmunity, T Cells

## Abstract

**Objective:**

Human leucocyte antigen (HLA)-B27 and endoplasmic reticulum aminopeptidase 1 (ERAP1) are strongly associated with ankylosing spondylitis (AS). ERAP1 is a key aminopeptidase in HLA class I presentation and can potentially alter surface expression of HLA-B27 free heavy chains (FHCs). We studied the effects of ERAP1 silencing/inhibition/variations on HLA-B27 FHC expression and Th17 responses in AS.

**Methods:**

Flow cytometry was used to measure surface expression of HLA class I in peripheral blood mononuclear cells (PBMCs) from patients with AS carrying different ERAP1 genotypes (rs2287987, rs30187 and rs27044) and in ERAP1-silenced/inhibited/mutated HLA-B27-expressing antigen presenting cells (APCs). ERAP1-silenced/inhibited APCs were cocultured with KIR3DL2CD3ε-reporter cells or AS CD4+ T cells. Th17 responses of AS CD4+ T cells were measured by interleukin (IL)-17A ELISA and Th17 intracellular cytokine staining. FHC cell surface expression and Th17 responses were also measured in AS PBMCs following ERAP1 inhibition.

**Results:**

The AS-protective ERAP1 variants, K528R and Q730E, were associated with reduced surface FHC expression by monocytes from patients with AS and HLA-B27-expressing APCs. ERAP1 silencing or inhibition in APCs downregulated HLA-B27 FHC surface expression, reduced IL-2 production by KIR3DL2CD3ε-reporter cells and suppressed the Th17 expansion and IL-17A secretion by AS CD4+ T cells. ERAP1 inhibition of AS PBMCs reduced HLA class I FHC surface expression by monocytes and B cells, and suppressed Th17 expansion.

**Conclusions:**

ERAP1 activity determines surface expression of HLA-B27 FHCs and potentially promotes Th17 responses in AS through binding of HLA-B27 FHCs to KIR3DL2. Our data suggest that ERAP1 inhibition has potential for AS treatment.

## Introduction

Ankylosing spondylitis (AS) is the prototype of the spondyloarthritis (SpA), a group of closely related chronic inflammatory diseases sharing clinical symptoms and strong genetic association with the human leucocyte antigen (HLA)-B27. The mechanism by which HLA-B27 confers disease susceptibility remains unclear. The canonical function of HLA-B27 is to form heterotrimers with β2-microglobulin (β2m) and antigenic peptides in the endoplasmic reticulum (ER), which then egress to the cell surface for CD8+ T cell recognition. However, lack of CD8+ T cells does not prevent disease in the HLA-B27-trangenic rat model of SpA, arguing against a primary role of CD8+ T cell activation by classical HLA-B27 in SpA.^1^ ^2^ We and others have shown the presence of HLA-B27 β2m-free heavy chains (FHCs) on the surface of peripheral blood mononuclear cells (PBMCs) from patients with SpA and HLA-B27-trangenic rats.^3–6^ The biological function of HLA-B27 FHCs is supported by its superior binding affinity, in comparison to classical HLA-B27, to the immunoregulatory receptors killer cell immunoglobulin-like receptor 3DL2 (KIR3DL2) and leucocyte immunoglobulin-like receptor B2 (LILRB2).^7^ ^8^ Importantly, binding of HLA-B27 FHCs to KIR3DL2 expressed by CD4+ T cells has been shown to promote the survival and proliferation of Th17 cells in AS.^9^ ^10^

The strong genetic association of AS with ER aminopeptidase 1 (ERAP1) has been reported by multiple studies in different ethnic groups.^11–17^ Five AS-associated ERAP1 single nucleotide polymorphisms (SNPs) were found: rs30187 (T/C, K528R), rs27044 (G/C, Q730E), rs2287987 (T/C, M349V), rs10050860 (C/T, D575N), rs17482078 (C/T, R725Q) (risk alleles and their corresponding amino acids are underlined). ERAP1 locates in the ER and trims peptides to optimal length (usually 8–10 amino acids) before their binding to major histocompatibility complex (MHC) class I molecules. Strikingly, ERAP1 polymorphisms only affect AS risk in HLA-B27-positive individuals, implying that ERAP1 contributes to AS pathogenesis by altering HLA-B27 function.^17^ Indeed, ERAP1 silencing or polymorphisms has been shown to alter the length and sequence of HLA-B27-bound peptides.^18^ ^19^ A recent study shows that AS-associated ERAP1 polymorphisms do not alter ER stress in patients with AS, arguing against the unfolded protein response theory.^20^ We hypothesised that ERAP1 might contribute to AS pathogenesis through altering cell surface HLA-B27 FHC expression.

To test this hypothesis, we studied the effect of ERAP1 silencing, inhibition and polymorphic variation on HLA-B27 FHC expression and Th17 function. Protective ERAP1 polymorphisms are associated with reduced HLA FHC expression in monocytes of patients with AS and HLA-B27-expressing antigen presenting cells (APCs). ERAP1 silencing or inhibition of APCs reduces HLA-B27 FHC expression, KIR3DL2 stimulation and Th17 responses. Finally, ERAP1 inhibition reduces HLA class I FHC expression and Th17 expansion in PBMCs from patients with AS.

## Materials and method

### Patients with AS

Heparinised venous blood was obtained from 56 HLA-B27-positive patients with AS fulfilling the modified New York criteria. Patient demographics are shown (see online supplementary table S1). Patients were assessed for disease activity using Bath AS Disease Activity Index (BASDAI), functional capacity using Bath AS Functional Index (BASFI) and spinal mobility using Bath AS Metrology Index (BASMI).

### Genotyping

DNA was prepared from peripheral blood cells using PureLink Genomic DNA Mini Kit (Life Technologies, UK). Three SNPs in the ERAP1 gene previously reported to be associated with AS, rs2287987 (T/C, M349V), rs30187 (T/C, K528R) and rs27044 (G/C, Q730E) were genotyped using functionally tested TaqMan Assays (Applied Biosystems, UK). Two additional AS-associated SNPs, rs10050860 (C/T, D575N), rs17482078 (C/T, R725Q), were not genotyped in this study. However, we found that they are in strong link disequilibrium with rs2287987 (T/C, M349V) in a set of 60 patients with AS (rs10050860: r^2^=1, D′=1; rs17482078: r^2^=0.956, D′=1, data not shown). This strong linkage was also found to be present in the general population using a public SNPs link disequilibrium calculation tool (rs10050860: r^2^=0.92, D′=1; rs17482078: r^2^=0.959, D′=1, SNP Annotation and Proxy Search, Broad Institute of Massachusetts Institute of Technology and Harvard).

### Cell lines

As described previously, HeLa.B27, C1R.B27 and mouse endoplasmic reticulum aminopeptidase associated with antigen processing (ERAAP)−/− fibroblasts (ERAAP−/− mFib.B27) were transfected to express HLA- HLA-B*27:05.^18^ ^21^ See online supplementary methods for cell culturing conditions.

### ERAP1 stable silencing and inhibition

As described previously, lentiviral ERAP1-shRNA was used to stably silence endogenous ERAP1 expression by HeLa.B27 and C1R.B27 cells.^18^ In the current study, DG013A was initially titrated at the concentration of 10 nM, 100 nM and 1000 nM. 1000 nM of DG013A was then used for ERAP1 inhibition.

### Construction and transfection of shRNA-resistant ERAP1 plasmids

See online supplementary methods.

### Isolation of PBMCs and CD4+ T cells

See online supplementary methods.

### Flow cytometry

PBMCs were blocked with Fc receptor blocking reagent (Miltenyi Biotec), stained for HLA class I FHCs with the HC-10 (IgG2a) and classical HLA-B27 complexes with ME-1 (IgG1). An APC-conjugated antimouse IgG antibody (Santa Cruz, USA) was used to detect HC-10 and ME-1. Monocyte, B cell and T cell were stained by CD14-PE (Miltenyi Biotec), CD19-Pacific Blue and CD3- PerCP-Cy5.5 (BioLegend, USA), respectively. Dead cells were excluded using Fixable Viability Dye eFluor 780 (Ebioscience, UK). In order to ensure unbiased comparison of FHC expression, all PBMC samples were stained and analysed in a single batch.

HeLa.B27, C1R.B27 and mFib.B27 cells were stained by HC-10 and ME-1 antibodies followed by APC-conjugated antimouse IgG antibody. Dead cells were excluded using LIVE/DEAD Fixable Violet Dead Cell Stain Kit (Life Technologies). BD LSRFortessa and Diva software were used. The latter converts channel value into fluorescence intensity using a logarithmic algorithm, therefore geometrical mean fluorescence intensity was used to quantify the intensity of HC-10 and ME-1 staining.

Intracellular cytokine staining of Th17/Th1 cells was carried out using BD Cytofix/Cytoperm kit (BD Bioscience, UK). Cells were stimulated with 100 ng/mL phorbol 12-myristate 13-acetate (PMA) (Sigma) and 1 µg/mL Ionomycin (Sigma) for 4 h in the presence of Golgi STOP and Golgi plug. After surface staining using CD3-BV605, CD4-APC and CD8-BV510 antibodies (Biolegend), cells were fixed and permeabilised, stained with interleukin (IL)-17A-FITC (Ebioscience) and interferon (IFN)-γ-AF700 (Biolegend). Dead cells were excluded using Fixable Viability Dye eFluor 780 (Ebioscience).

### KIR3DL2CD3ε reporter cell assay

The KIR3DL2CD3ε reporter cells secreting IL-2 have been previously described.^9^ ^10^ See online supplementary methods for details.

### Coculture of CD4+ T cells with APCs

About 100 000 CD4+ T cells were cocultured with 5000 HeLa.B27/C1R.B27 cells in 50 µL/200 µL R10 supplemented with 1 pg/mL staphylococcal enterotoxin B (SEB) and 50 ng/mL IL-2. Supernatants were harvested on day 3 for IL-17A ELISA (Ebioscience) cells stained on day 6 for Th17.

### ERAP1 inhibition of AS PBMCs

About 100 000 AS PBMCs were cultured overnight in 100 µL R10 with/without the addition of DG013A (1000 nM), stained for classical HLA-B27 and FHC surface expression. For Th17 responses, 500 000 AS PBMCs were cultured in 200 µL R10 with the addition of SEB (1 pg/mL), IL-2 (50 IU/mL) and DG013A (1000 nM) for 6 days. Supernatants were collected on day 3 for IL-17A ELISA, cells stained on day 6 for Th17. DG013A was replenished daily, SEB and IL-2 on day 3.

### Statistics

**Figure 1:**
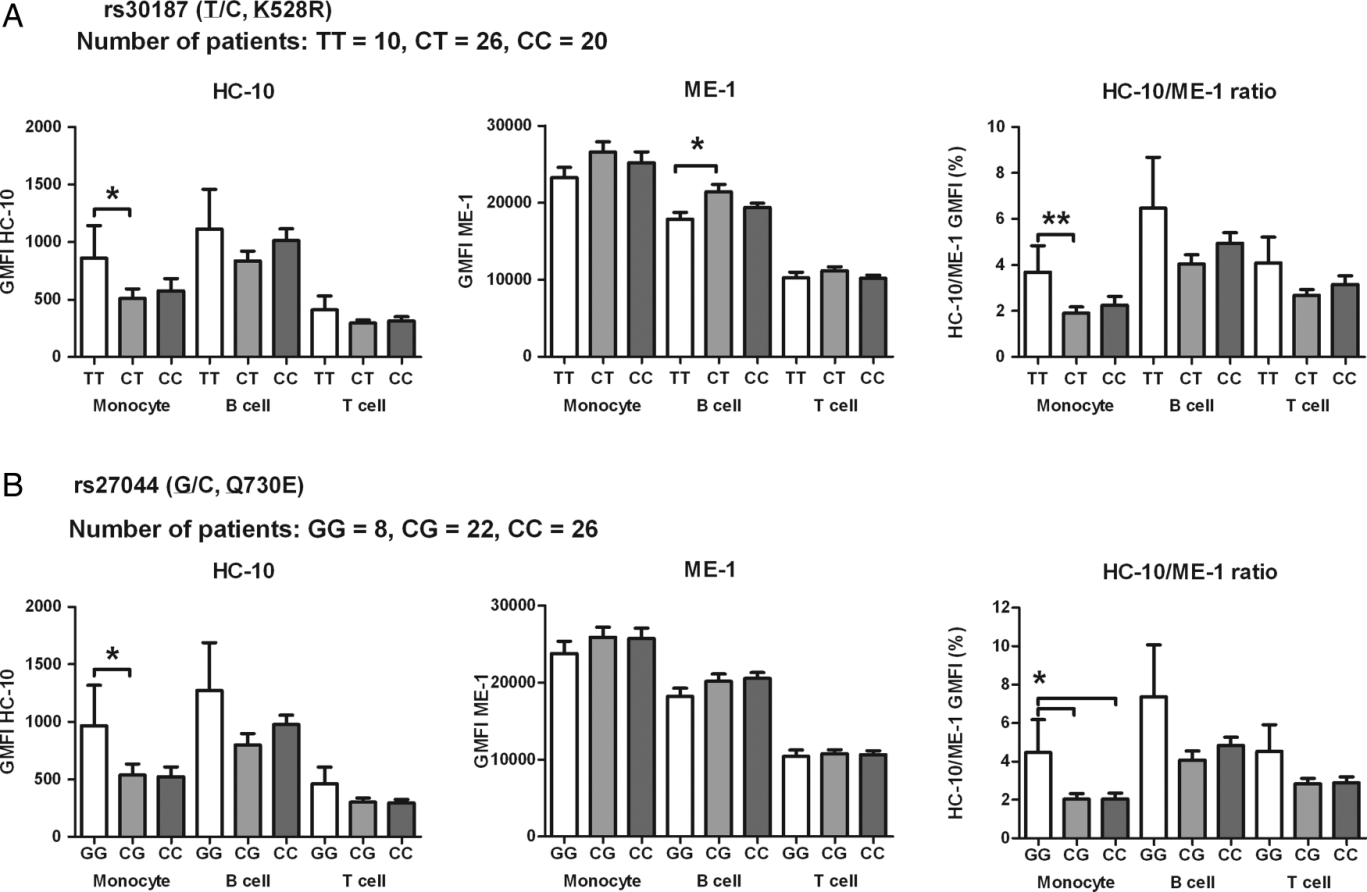
Patients with ankylosing spondylitis (AS) carrying protective endoplasmic reticulum aminopeptidase 1 variants, K528R or Q730E, have lower monocyte human leucocyte antigen (HLA) class I free heavy chain (FHC) expression. HC-10 antibody (HLA class I FHCs) and ME-1 antibody (classical β2m-associated HLA-B27, B7, B42, B67, B73 and Bw22) staining of CD14+ monocyte, CD19+ B cell and CD3+ T cell from peripheral blood mononuclear cells in patients with AS are shown. HC-10 staining, ME-1 staining and HC-10/ME-1 are compared between patients with AS with different genotypes of rs30187 (TT, CT, CC) in (A) and rs27044 (GG, CG, CC) in (B). Risk alleles and corresponding amino acids are underlined. Results are expressed as mean and SE of mean, p value was determined by Mann-Whitney test (*p<0.05, **p<0.01). Insufficient HLA-B27+ healthy controls were available for comparison.

**Figure 2:**
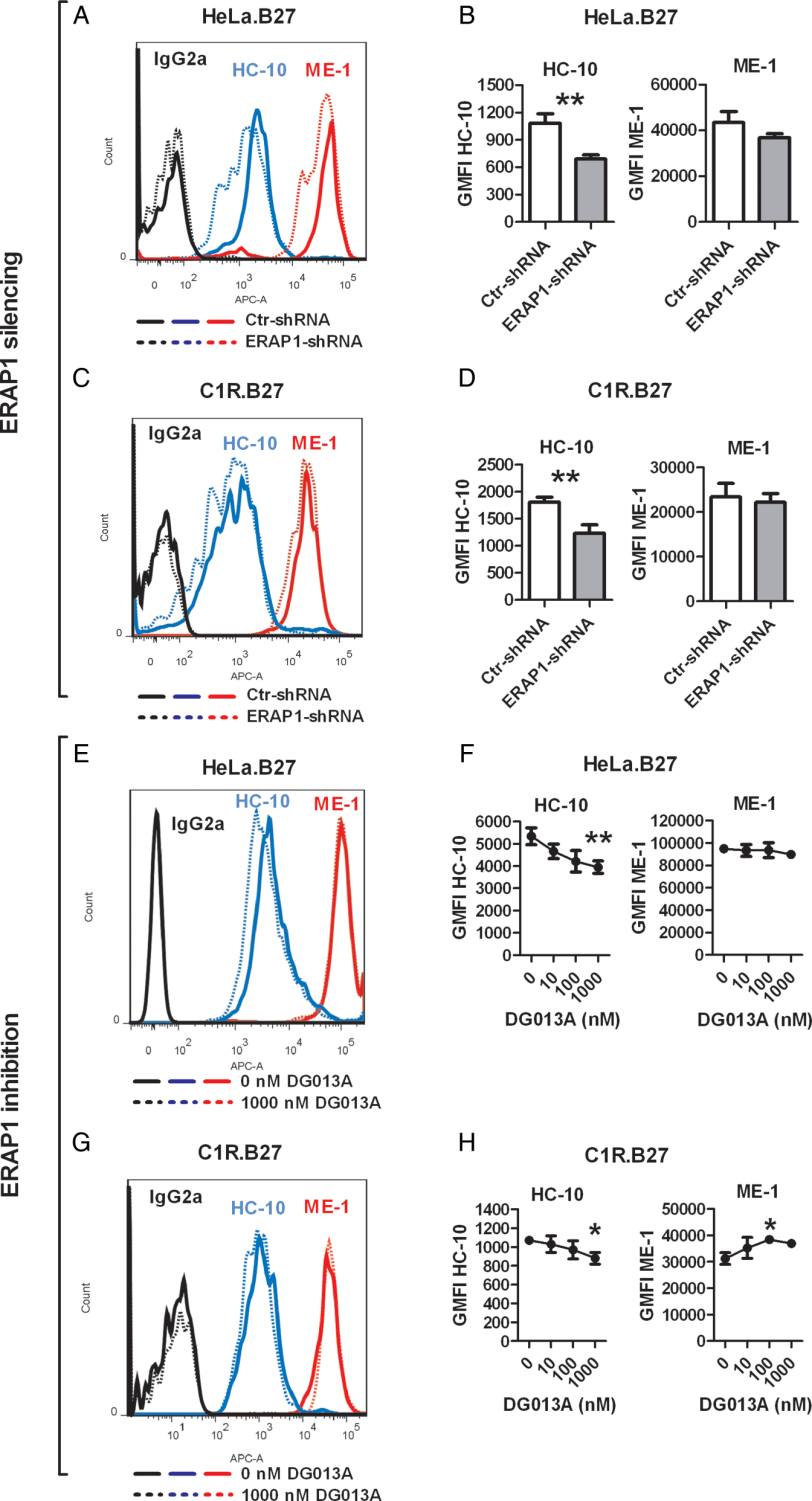
Endoplasmic reticulum aminopeptidase 1 (ERAP1) silencing or inhibition reduces surface human leucocyte antigen (HLA)-B27 free heavy chain (FHC) expression by HeLa.B27 or C1R.B27 cells. The expression of HLA-B27 FHCs (HC-10 antibody) and classical HLA-B27 complexes (ME-1 antibody) surface expression are compared between ctr-shRNA and ERAP1-shRNA-transduced HeLa.B27 cells (A) and C1R.B27 cells (C). Summaries are shown in (B) and (D). Neither the IgG2a (isotype control for HC-10, data shown here) nor IgG1 (isotype control for ME-1, data not shown) stains HeLa.B27 and C1R.B27 cells. Representative stains of HeLa.B27 cells (E) and C1R.B27 cells (G) treated with 1000 nM DG013 are shown. Summaries of DG013A titration (0 nM, 10 nM, 100 nM, 1000 nM) on HeLa.B27 cells and C1R.B27 cells are shown in (F) and (H). All experiments were repeated three times. Results are expressed as mean and SD. p Value was determined using unpaired two-tailed t test (*p<0.05, **p<0.01).

**Figure 3:**
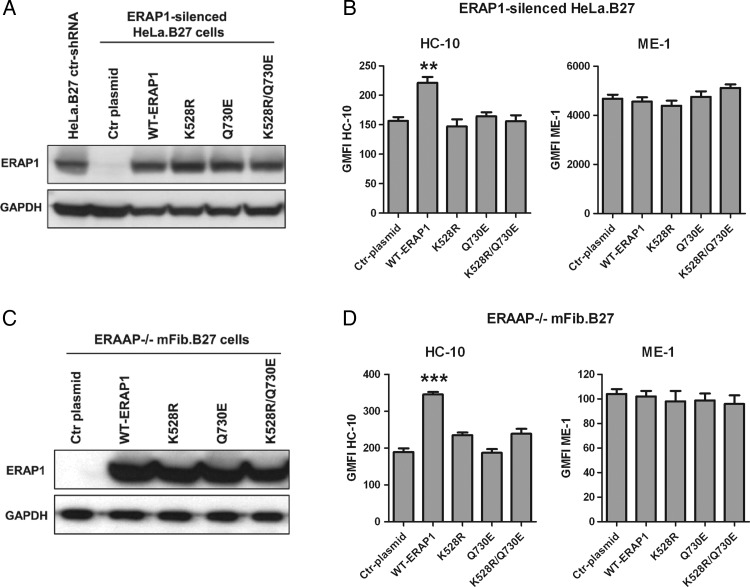
Wild type endoplasmic reticulum aminopeptidase 1 (ERAP1), but not the protective variants K528R or Q730E, increases cell surface human leucocyte antigen (HLA)-B27 free heavy chain (FHC) expression. WT-ERAP1 and protective ERAP1 variants (K528R, Q730E, K528R/Q730E) were transiently expressed in ERAP1-silenced HeLa.B27 cells and ERAAP-knockout mFib.B27 cells. (A and C) Western blot showing that ERAP1 variants have been expressed at similar levels. The expression of HLA-B27 FHCs (HC-10 antibody) and classical HLA-B27 complexes (ME-1 antibody) surface expression are compared between HeLa.B27 cells (B) and mFib.B27 cells (D) expressing different ERAP1 variants. Both experiments were repeated three times. Results are expressed as mean and SD. p Value was determined using one way ANOVA (**p<0.01, ***p<0.001).

**Figure 4:**
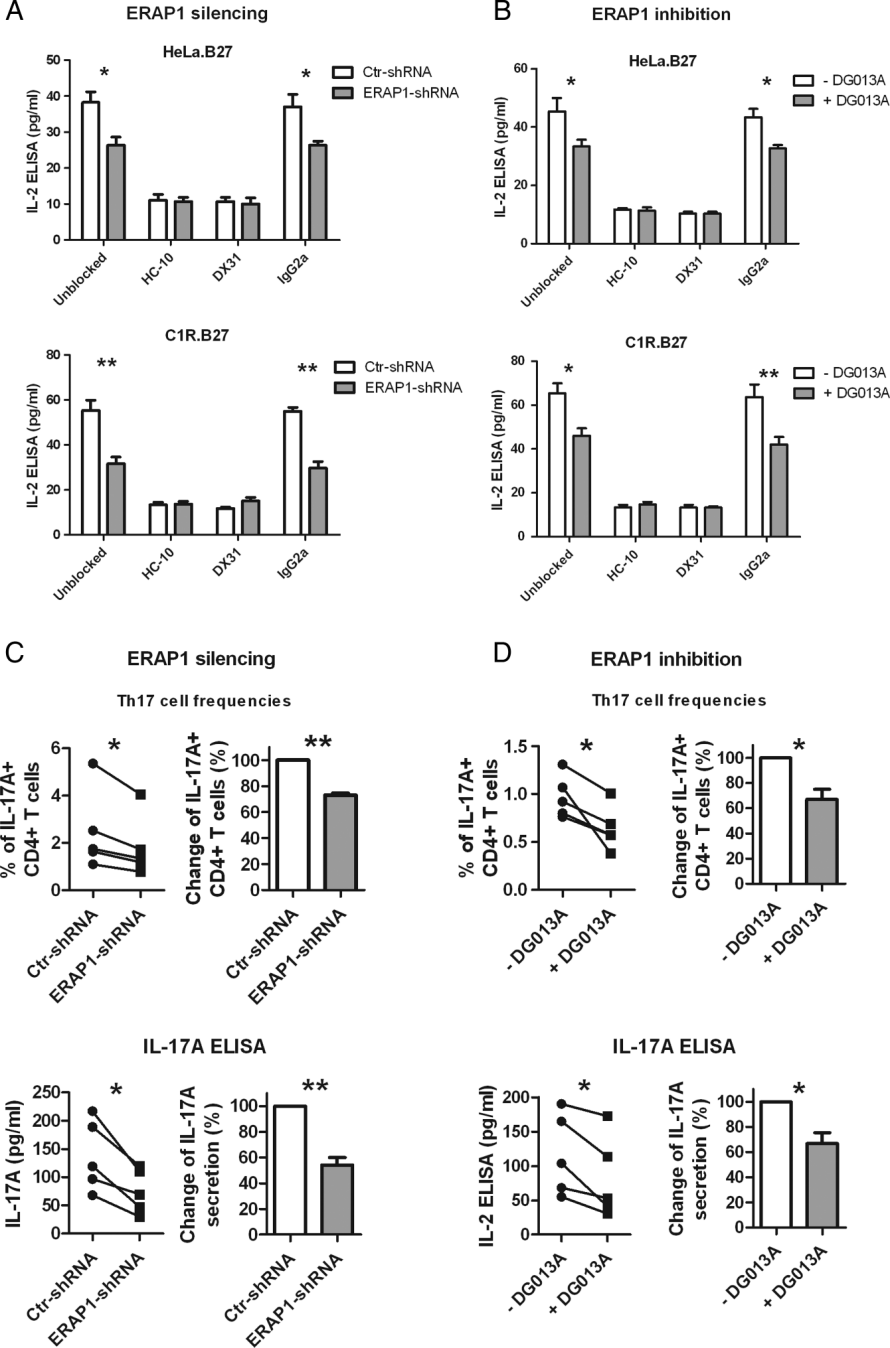
Endoplasmic reticulum aminopeptidase 1 (ERAP1) silencing or inhibition of human leucocyte antigen (HLA)-B27-expressing antigen presenting cells (APCs) reduces interleukin (IL)-2 production by KIR3DL2CD3ε-expressing reporter cells, and inhibits Th17 cell expansion and IL-17A production by CD4+ T cells from patients with ankylosing spondylitis (AS). The effect of ERAP1 silencing (A) and inhibition (B) in APCs on IL-2 production by KIR3DL2CD3ε-expressing reporter cells are shown. IL-2 ELISA was used to measure the ligation of KIR3DL2 by HLA-B27 free heavy chains. HC-10 or DX31 blocked the production of IL-2, but not isotype control antibody. The effect of ERAP1 silencing (C) and inhibition (D) in APCs on Th17 expansion and IL-17A production are also shown. HLA-B27-expressing APCs were cocultured with CD4+ T cells from patients with AS (n=5) at the presence of 50 U/mL IL-2 and 1 pg/mL staphylococcal enterotoxin B. Supernatant was collected for IL-17A ELISA at day 3, cells were harvested for Th17 cell staining at day 6. Experiments in (A and B) were repeated three times. Results are expressed as mean and SD (A and B) or SE (C and D). p Value was determined using unpaired two-tailed t test in (A and B) and paired two-tailed t test in (C and D) (*p<0.05, **p<0.01).

**Figure 5:**
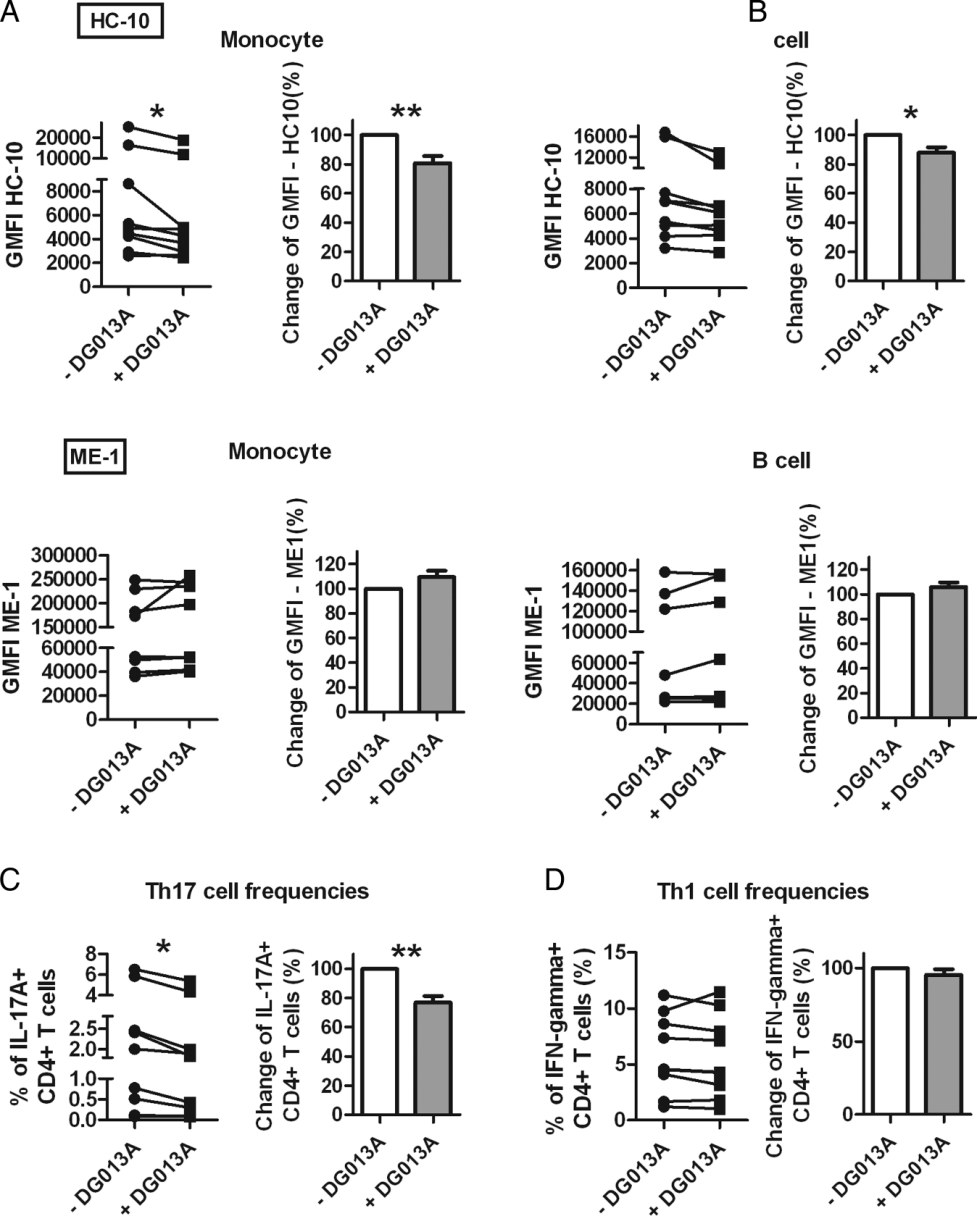
Endoplasmic reticulum aminopeptidase 1 inhibition reduces peripheral blood mononuclear cell (PBMC) surface human leucocyte antigen (HLA) class I free heavy chain (FHC) expression and Th17 expansion in patients with ankylosing spondylitis (AS). (A) PBMCs from patients with AS (n=9) were cultured with 1000 nM DG013A for 16 h. HLA class I FHCs (HC-10 antibody) and classical HLA-B27 (ME-1 antibody) surface expression by CD14+ monocyte, CD19+ B cell from PBMCs in patients with AS are shown. (B) PBMCs from patients with AS (n=9) were cultured with 1000 nM DG013A for 6 days in the presence of 50 U/mL IL-2, 1 pg/mL staphylococcal enterotoxin B. Supernatant was collected for IL-17A ELISA at day 3, cells were harvested for Th1 and Th17 cell staining at day 6. For both experiments, patients with AS with high basal HC-10 expression were chosen. Results are expressed as mean and SE. p Value was determined using paired two-tailed t test (*p<0.05, **p<0.01).

Results are expressed as mean and SE of mean (figures 1, 4C, D and 5) or SD (other figures). The statistical significance of differences between means was assessed using Mann-Whitney test (figure 1), unpaired two-tailed t test (figures 2 and 4A, B), one way Analysis of variance (ANOVA) (figure 3) or paired two-tailed t test (figures 4C, D and 5). A p value <0.05 was considered statistically significant.

## Results

### Protective ERAP1 variants are associated with reduced HLA class I FHC expression by AS monocytes

We first investigated the effect of ERAP1 polymorphisms on FHC expression in PBMCs from HLA-B27-positve patients with AS. Three AS-associated ERAP1 SNPs were studied: rs30187 (T/C, K528R), rs27044 (G/C, Q730E) and rs2287987 (T/C, M349V). Risk alleles and their corresponding amino acids are underlined. No significant difference in the age, BASDAI, BASFI, BASMI was present between genotypical groups (see online supplementary table S1). Figure 1 shows that monocytes from patients carrying AS protective alleles of rs30187 or rs27044 (528R or 730E) express significantly lower absolute (HC-10) and relative (HC-10/ME-1 ratio) levels of HLA class I FHCs. A similar but non-significant trend was seen in B cells and T cells. No significant difference was found between patients carrying risk and protective alleles of rs2287987 (see online supplementary figure S1).

### ERAP1 silencing or inhibition reduces HLA-B27 FHC expression

The K528R and Q730E ERAP1 variants, corresponding to protective alleles of rs30187 and rs27044 respectively, have been shown to reduce enzyme activity.^17^ ^18^ ^22–24^ We therefore explored the effects of ERAP1 silencing on HLA-B27 FHCs expression. Lentiviral ERAP1-shRNA was used to stably suppress the expression of endogenous ERAP1 by HeLa.B27 and C1R.B27 cells (85–90% suppression, see online supplementary figure S2). As shown in figure 2A–D, ERAP1 silencing led to a 30–40% reduction of HLA-B27 FHC expression by HeLa.B27 and C1R.B27 cells. Unlike the untransfected C1R cells, which express low levels of MHC class I molecules and bind only weakly to HC-10 antibody, untransfected HeLa cells could be stained by HC-10 (see online supplementary figure S3). In order to exclude the possibility that the reduced HC-10 staining following ERAP1-silencing in HeLa.B27 cells was due to downregulation of other MHC class I, we silenced ERAP1 in untransfected HeLa. This did not decrease HC-10 staining (see online supplementary figure S4). In addition to ERAP1 silencing, we also examined the effect of ERAP1 inhibition on HLA-B27 FHC expression using a recently identified compound, DG013A.^25^ DG013A is a phosphinic pseudopeptide that binds to the catalytic site of ERAP1. It also inhibits ERAP2 and leucyl/cystinyl aminopeptidase (LNPEP) (both AS-associated).^25^ ^26^ Figure 2E–H show that DG013A decreased FHC expression by HeLa.B27 and C1R.B27 cells in a dose-dependent manner, without reducing ME-1 levels. Moreover, ERAP1 silencing or inhibition also significantly reduced the staining by HD-6, an antibody raised against HLA-B27 homodimers (see online supplementary figure S5).^10^ ^27^

### Expression of wild type (WT)-ERAP1, but not AS protective variants, in ERAP1-silenced HeLa.B27 and ERAAP−/− mFib.B27 cells increases FHC expression

To further confirm the role of ERAP1 in determining HLA-B27 FHC expression, we next introduced WT-ERAP1 into ERAP1-silenced HeLa.B27 and ERAAP−/− mFib.B27 cells. ShRNA-resistant ERAP1 plasmids encoding WT and variants were constructed by introducing multiple synonymous mutations in the shRNA-targeting sequence (see online supplementary figure S6A). Online supplementary figure S6B shows that ERAP1 was successfully expressed in ERAP1-silenced HeLa.B27 cells using ShRNA-resistant ERAP1 plasmids but not unmutated WT-ERAP1. Expression of WT-ERAP1 significantly upregulated the expression of HLA-B27 FHCs in ERAP1-silenced HeLa.B27 (figure 3A, B) and ERAAP−/− mFib.B27 cells (figure 3C, D) (see online supplementary figure S7 for representative stains). Transfection using WT-ERAP1 without synonymous mutations did not affect the expression of surface FHCs, further confirming that the increase of FHC expression is due to introduction of ERAP1 (see online supplementary figure S6C).

Figure 3 also shows that, in ERAP1-silenced HeLa.B27 and ERAAP−/− mFib.B27 cells, the K528R, Q730E and K528R/Q730E variants did not upregulate FHCs. The double variant was studied because of the genetic linkage between K528R and Q730E (r^2^=0.734, D′=1, SNP Annotation and Proxy Search). Notably, no significant difference in ERAP1 expression levels was observed between ERAP1 variants (figure 3A, C).

### ERAP1 silencing or inhibition of HLA-B27-expressing APCs reduces ligation of KIR3DL2 by HLA-B27 FHCs and Th17 responses from AS CD4+ T cells

The best characterised function of HLA-B27 FHCs is their interaction with KIR3DL2. HLA-B27 FHCs bind to KIR3DL2 more strongly than classical HLA-B27 molecules, and stimulate the survival, proliferation and IL-17A production of KIR3DL2^+^ CD4+ T cells from patients with AS.^9^ We therefore asked if ERAP1 suppression/inhibition affects functional recognition of HLA-B27-expressing APCs by KIR3DL2. When HeLa.B27 or C1R.B27 cells were cocultured with KIR3DL2CD3ε reporter cells, ERAP1 silencing (figure 4A) or inhibition (figure 4B) reduced the activation of this receptor (reflected by IL-2 production). Blocking using either HC-10 or DX31 (specific for KIR3DL2) antibodies reduced IL-2 to basal levels, confirming that IL-2 secretion in this assay is due to the interaction of HLA-B27 FHCs and KIR3DL2.

We next investigated whether ERAP1 silencing or inhibition of APCs affected Th17 expansion when the APCs were cocultured with CD4+ T cells from patients with AS. Differentiation of Th17 cells and secretion of IL-17A were reduced when ERAP1 of APCs was silenced (figure 4C) or inhibited (figure 4D).

### ERAP1 inhibition reduces surface HLA class I FHCs and Th17 cell frequency of PBMCs in patients with AS

We lastly examined the potential of ERAP1 inhibition to modulate FHC expression and Th17 expansion in PBMCs from patients with AS. Figure 5A shows that overnight treatment of PBMCs using DG013A decreased HLA class I FHC expression by monocytes and B cells. No effect was observed on T cells (see online supplementary figure S8A). ERAP1 inhibition also decreased the Th17 expansion from AS PBMCs after 6 days (figure 5B), without significantly reducing the frequency of IFN-γ-producing Th1 cells (see online supplementary figure S8B).

## Discussion

In this study we show that ERAP1 plays an important role in determining the expression levels of cell surface HLA-B27 FHCs. Genetic silencing and chemical inhibition of ERAP1 led to similar reduction of HLA-B27 FHC expression, indicating that the effect is specific. By contrast, the introduction of wild-type ERAP1 into ERAP1-silenced or ERAAP-knockout cells increased FHC expression. Increased monocyte FHC expression in patients with AS has been described previously,^4^ ^6^ but in these studies ERAP1 was not genotyped. We show for the first time that patients with AS carrying protective allelic variants of ERAP1, 528R and 730E, have reduced monocyte HLA class I FHC expression. Our result provides an immunological explanation for the low AS risk conferred by these two ERAP1 variants. The fact that the 528R and 730E variants, similar to ERAP1 silencing and inhibition, have downregulating effect on HLA-B27 FHCs suggests that they are effectively loss-of-function variants. This is supported by our finding that introduction into ERAP1-silenced or ERAAP−/− cell lines of these variants, singly or in combination, does not upregulate HLA-B27 FHC expression. This is also in accordance with previous in vitro studies using synthetic peptides and cell-based HLA-B27 peptidome studies.^17^ ^19^ ^22–24^ ^28^ We did not observe a significant effect for the 349V variant, noting that its genetic association with AS is much weaker.^13^ ^17^ Notably, although all patients with AS in this study were HLA-B27-positive, the contribution of other HLA class I molecules to PBMC FHC expression could not be excluded. Our results differ in some aspects from the findings of Haroon *et al*,^29^ which found that 730E increased monocyte FHC levels in patients with AS and 528R had little effect. They also found that ERAP1 siRNA silencing of C1R.B27 cells did not alter cell surface FHC expression. In the current study we have achieved more efficient and stable ERAP1 silencing using shRNA, and studied the HeLa.B27 cells in addition to C1R.B27 cells. Moreover, in addition to monocytes of patients with AS , we showed the reduction of FHC expression by the 528R and 730E mutations in ERAP1-reconstituted cell lines. Finally, we also studied the effect of ERAP1 inhibition on FHC expression in cell lines and AS PBMCs.

We also show that ERAP1 silencing or inhibition of APCs has functional effects on KIR3DL2 stimulation and Th17 cell expansion. Silencing or inhibition of HeLa.B27 and C1R.B27 cells reduced recognition by a KIR3DL2 reporter cell line by approximately 30%, similar to the amount of reduction in FHC surface expression. When CD4+ T cells in patients with AS were cultured with APCs, ERAP1 silencing or inhibition reduced Th17 expansion and IL-17A secretion. These finding suggest that ERAP1 inhibition might have therapeutic benefit in AS.

Our findings might at first appear to be difficult to reconcile with our previous finding that ERAP1 silencing increases the length of peptides (11–13 polymers) bound to HLA-B27.^18^ However, these long peptides stabilised classical surface HLA-B27 molecules just as well as 9 nonamer HLA-B27 epitopes in T2 HLA-B27 stabilisation assays,^18^ suggesting that HLA-B27 complexes loaded with longer peptide ligands are in fact as stable as those carrying 9–10 polymer epitopes. These longer peptides are potentially optimal ligands for classical HLA-B27 complex formation, and are enriched when ERAP1 is silenced. Thus suppression of ERAP1 might increase the overall pool of high affinity peptide ligands available for binding to HLA-B27. This could explain our finding that surface HLA-B27 FHC expression is reduced by ERAP1 silencing or inhibition. Therefore, ERAP1 might contribute to disease by actively destroying HLA-B27-binding peptide epitopes. Indeed, WT-ERAP1 has been shown to more rapidly destroy multiple HLA-B27-destined peptides than loss-of-function ERAP1 variants.^28^ Recently published data has suggested that ERAP1 allotypes/haplotypes with abnormally high or low peptide trimming activity may predispose to AS by limiting the supply of optimal peptides to HLA-B27 and therefore adversely affecting its conformational stability.^30^ Alternatively, ERAP1 may contribute to a chaperone activity within the ER resulting in accelerated egress of HLA-B27 FHCs and classical HLA-B27 molecules. As shown in figure 2A–D, in addition to reducing HLA-B27 FHC surface expression, ERAP1 silencing of APCs has a trend, although not significant, to downregulate surface classical HLA-B27.

Notably DG013A is a potent inhibitor for ERAP2 and LNPEP as well as ERAP1.^25^ While we cannot exclude effects on these aminopeptidases, it is important to note that ERAP2 and LNPEP are also associated with AS.^26^ Any off-target effect could thus potentially be beneficial in AS treatment. Moreover, the reduction of HLA-B27 FHC levels by DG013A was not seen in 221.B27 cells (data not shown) and reduction of PBMC HLA class I FHC expression was not seen for two out of nine patients with AS (figure 5A). ERAP1 inhibitors with higher selectivity and/or potency are worth investigating.

Overall, our data show that ERAP1 plays a critical role in determining levels of HLA-B27 FHC expression and Th17 responses in AS. ERAP1 silencing or inhibition reduces HLA-B27 FHC expression. Disease-protective ERAP1 alleles are associated with lower FHC expression levels in reconstituted cell lines and patients with AS. We also provide the first evidence that ERAP1 inhibition may suppress Th17 response in AS. Therefore, ERAP1 inhibition could potentially be used for therapy in AS.

## Supplementary Material

Web figures

Web methods

Web table
