# Supplementary material for: Silencing or inhibition of endoplasmic reticulum aminopeptidase 1 (ERAP1) suppresses free heavy chain expression and Th17 responses in ankylosing spondylitis
Source: Ann Rheum Dis. 2015 Jun 30;75(5):916–23. doi: 10.1136/annrheumdis-2014-206996 (PMC4853590; doi:10.1136/annrheumdis-2014-206996)
Supplement: Web figures [file annrheumdis-2014-206996-s1.pdf]

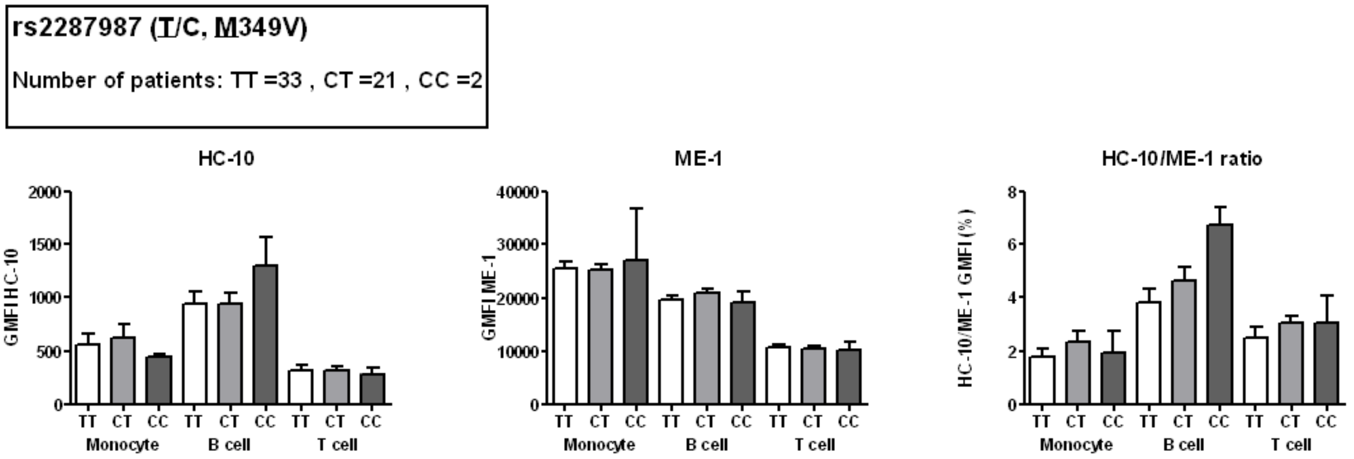

Figure S1 AS patient PBMC HLA class I free heavy chain expression is not affected by ERAP1 variant, M349V. HLA class I FHCs (HC-10 antibody) and classical HLA-B27 complexes (ME-1 antibody) surface expression by CD14+ monocyte, CD19+ B cell and CD3+ T cell from AS patient PBMCs are shown. HC-10 staining, ME-1 staining and HC-10/ME-1 are compared between AS patients with different genotypes of rs2287987 (TT, CT, CC). Risk allele and corresponding amino acid are underlined. Results are expressed as mean and standard error of mean, P value was determined by Mann-Whitney test.

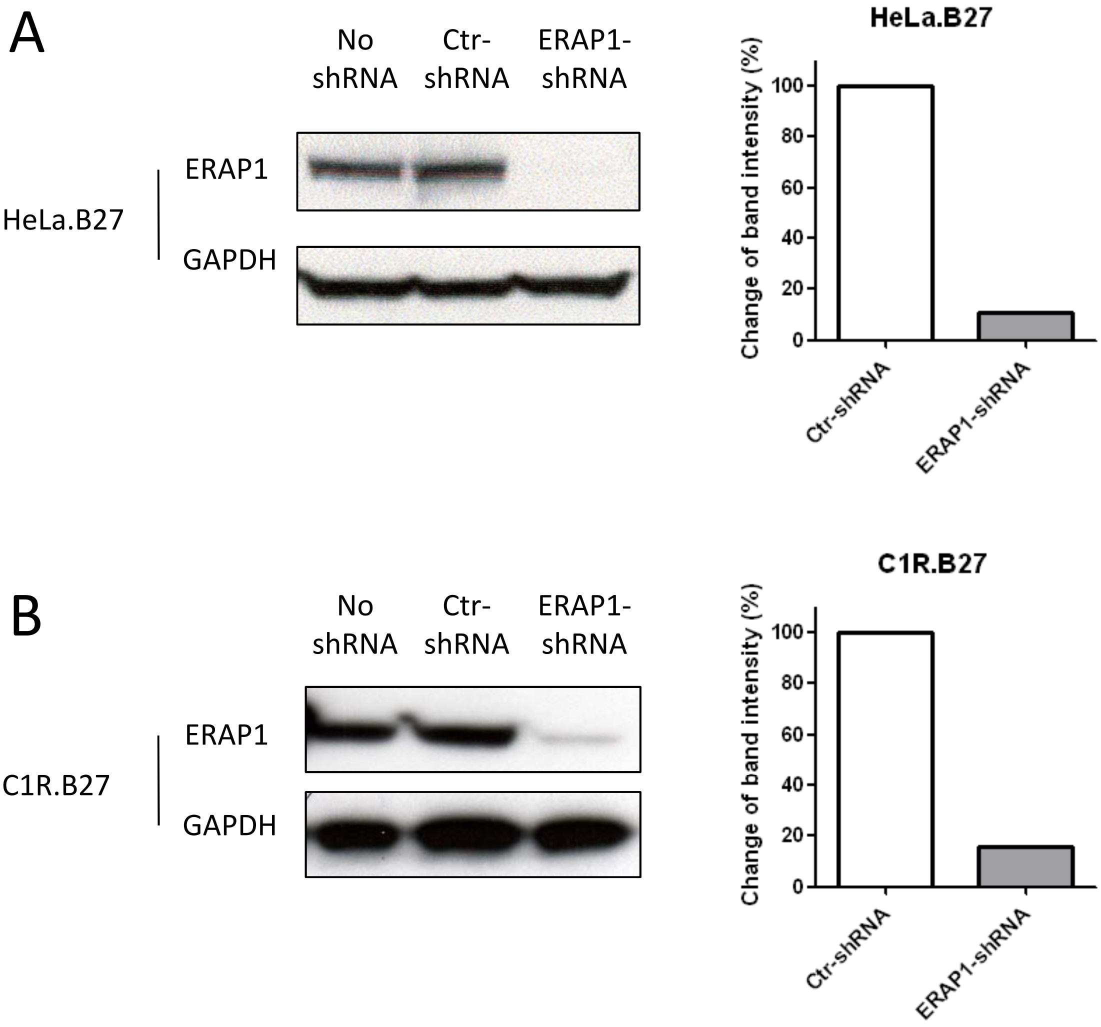

Figure S2. Stable silencing of endogenous ERAP1 expression in HeLa.B27 (A) and C1R.B27 (B) cells using lentiviral ERAP1-shRNA. Protein band intensity was calculated using Image-J.

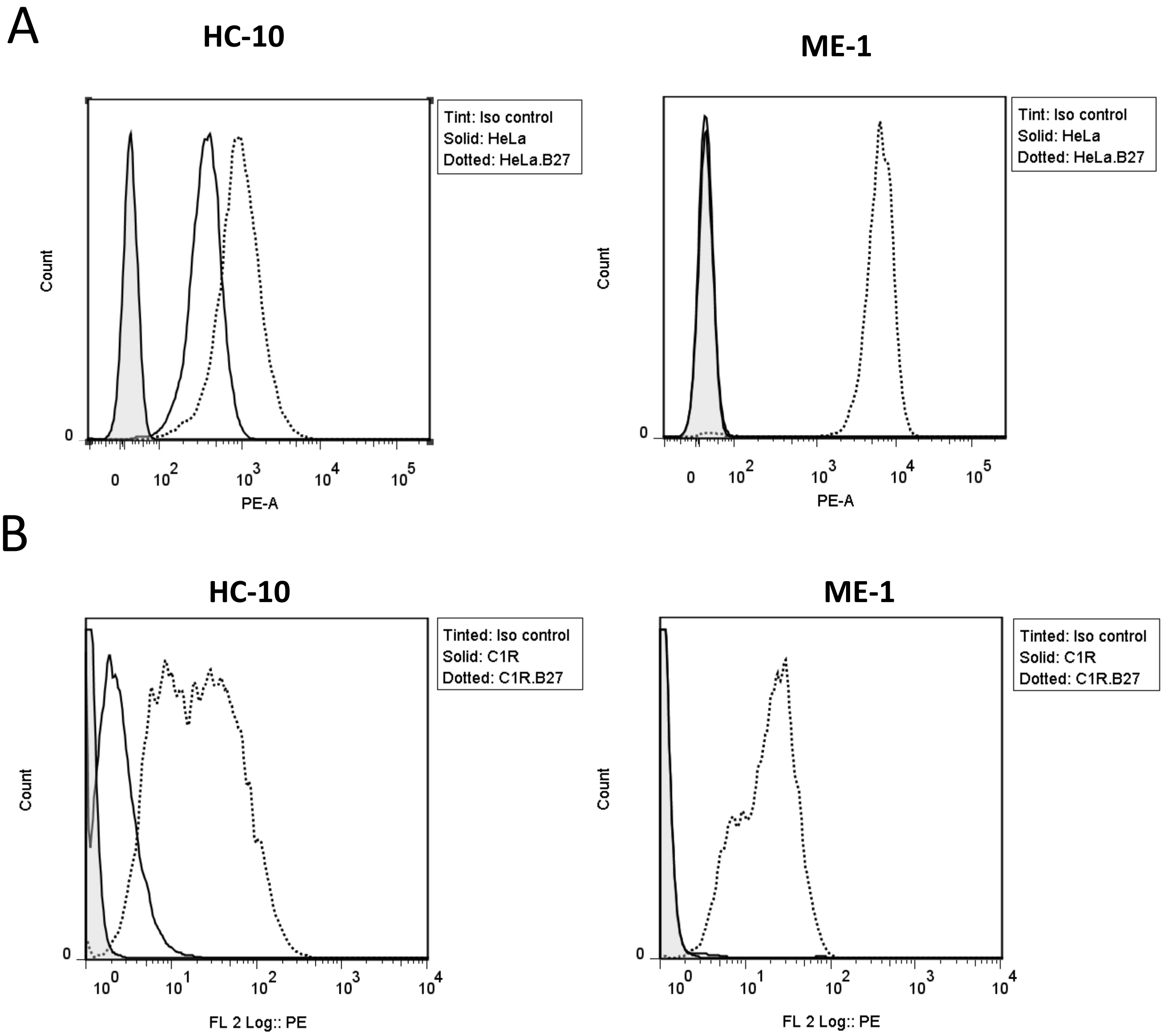

Figure S3 ME1 stains only HeLa.B27 and C1R.B27 cells, HC-10 stains HeLa and C1R but to a lesser extent compared to cells expressing B27.

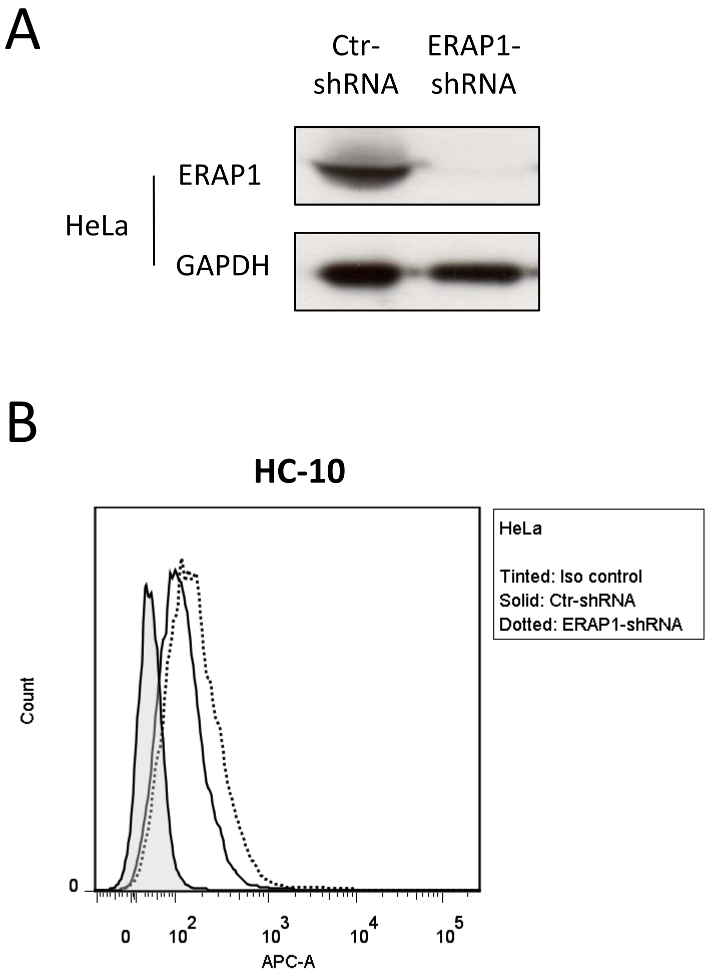

Figure S4. ERAP1 silencing does not reduce MHC class I free heavy chain expression (HC-10) by HeLa cells (n=2).

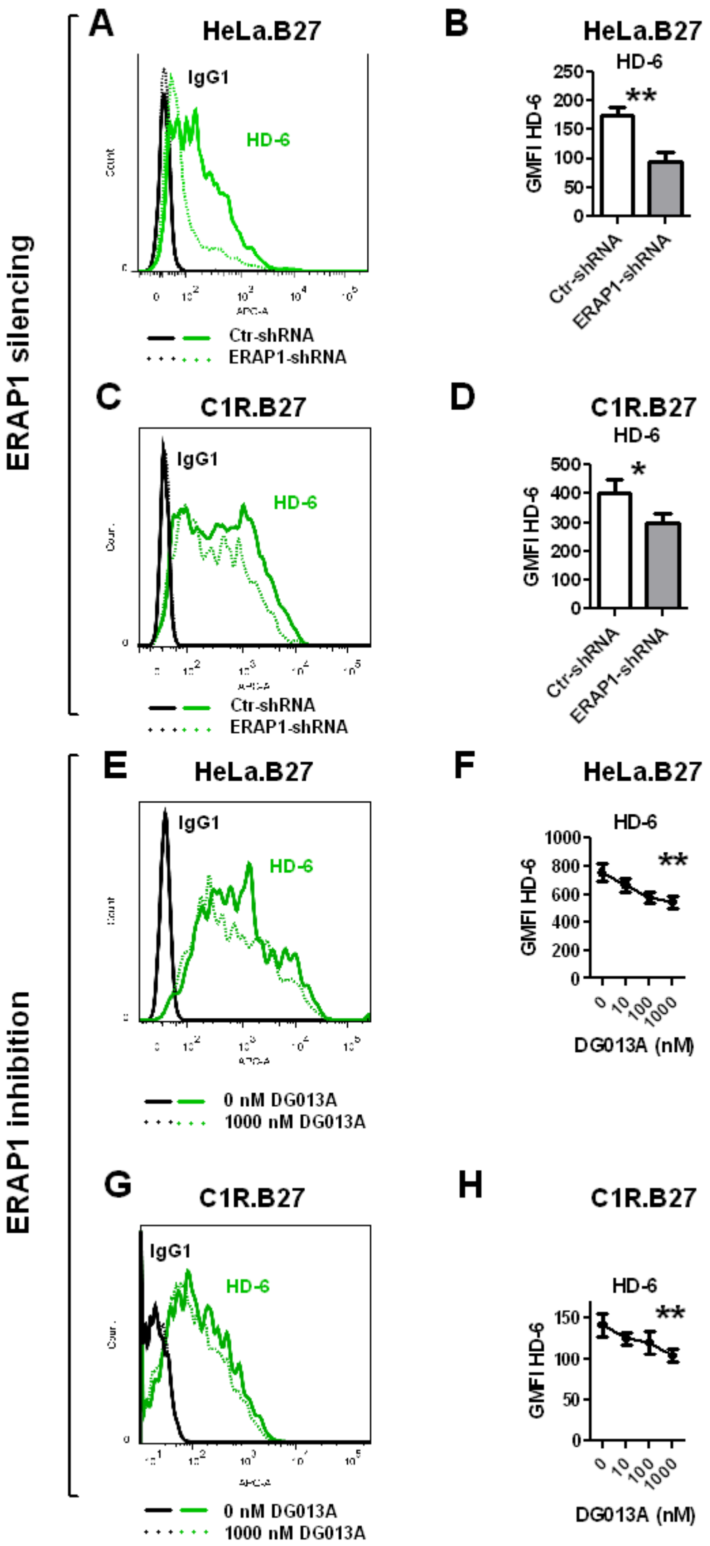

Figure S5 ERAP1 silencing or inhibition reduces surface HD-6-reactive molecule by HeLa.B27 or C1R.B27 cells. Representative HD-6 staining of ctr-shRNA and ERAP1-shRNA-transduced HeLa.B27 cells (A) and C1R.B27 cells (C) are shown; corresponding summary is shown in (B) and (D). Representative stains of HeLa.B27 cells (E) and C1R.B27 cells (G) treated with 1000 nM DG013 are shown. Summaries of DG013A titration (0, 10, 100, 1000 nM) on HeLa.B27 cells and C1R.B27 cells are also shown in (F) and (H). All experiments were repeated three times. Results are expressed as mean and standard deviation. P value was determined using unpaired two-tailed t-test (* = P<0.05, ** = p<0.01).

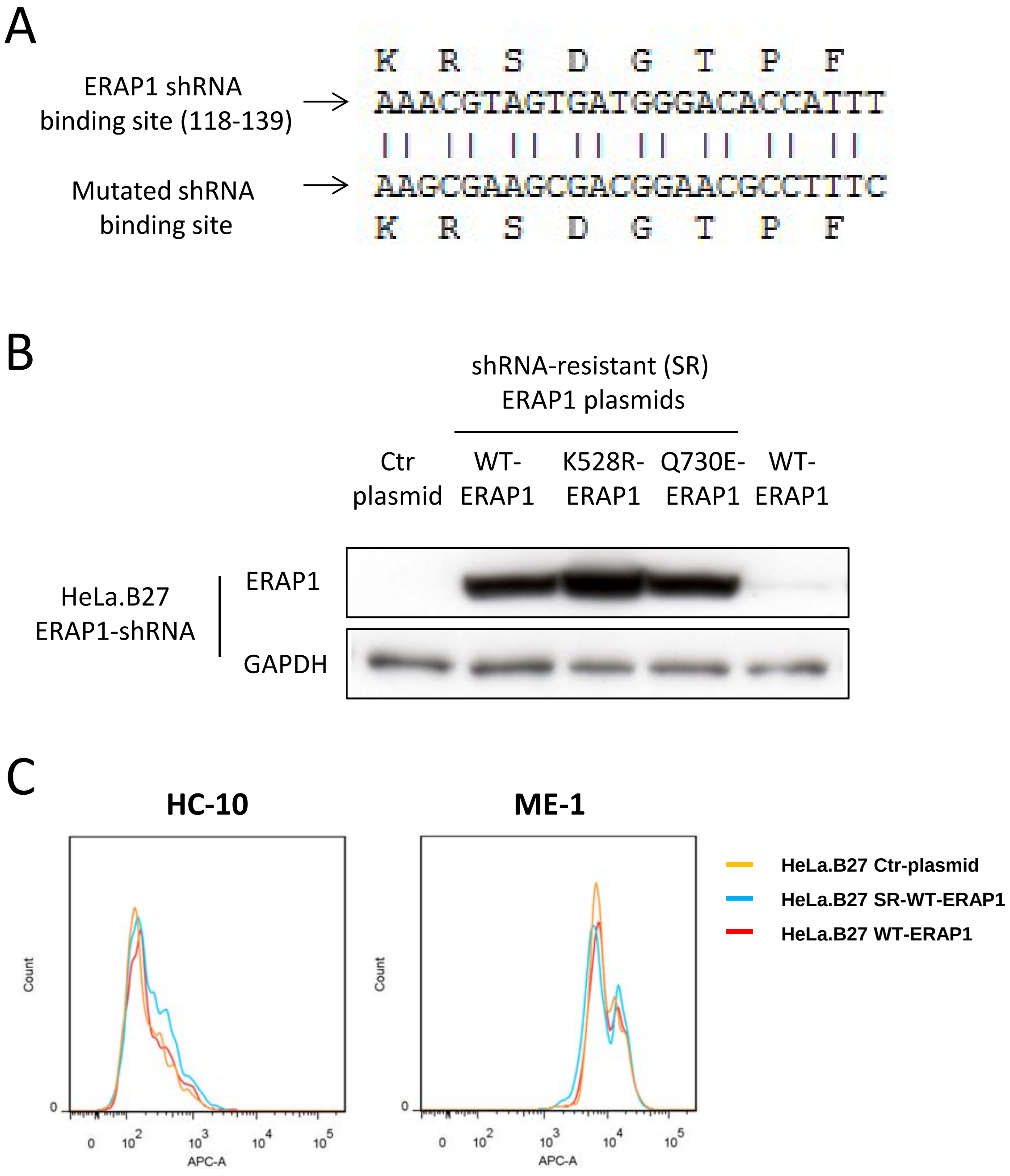

Figure S6 Expression of ERAP1 in ERAP1-silenced cells using shRNA-resistant (SR) ERAP1 plasmid. (A) Mutation of ERAP1 shRNA binding site. (B) ERAP1 is expressed in ERAP1-silenced cells only by transfection using SR-ERAP1 variants, not un-mutated WT-ERAP1. (C) HLA-B27 free heavy chain expression (HC-10) is altered by transfection using SR-WT-ERAP1, but not WT-ERAP1.

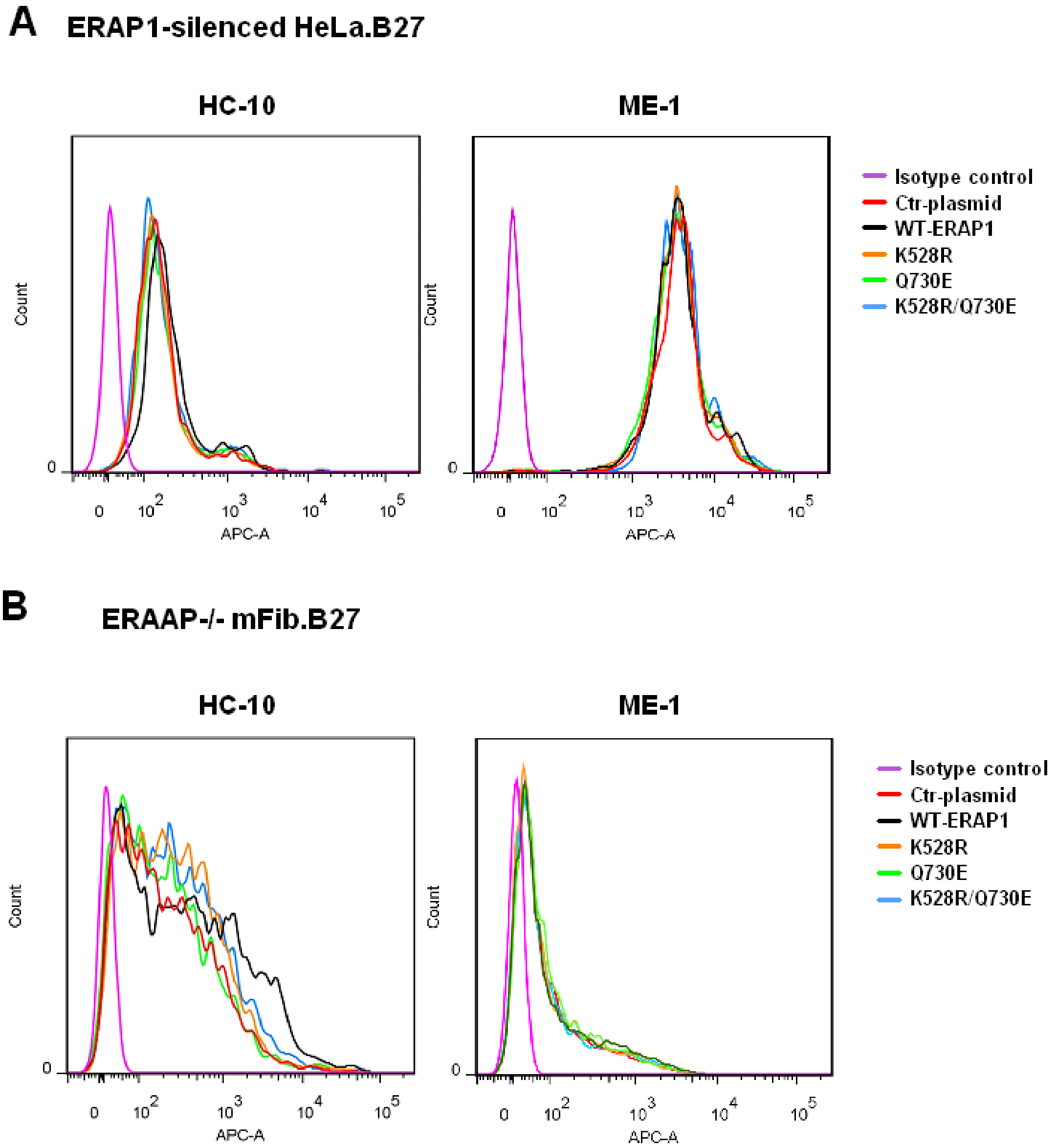

Figure S7 Wild type ERAP1, but not the protective variants K528R or Q730E, increases cell surface HLA-B27 free heavy chain expression. Representative HC-10 and ME-1 stains of ERAP1-silenced HeLa.B27 cells (A) and ERAAP-/- mFib.B27 cells (B) transiently expressing WT-ERAP1 and protective ERAP1 variants (K528R, Q730E, K528R/Q730E)

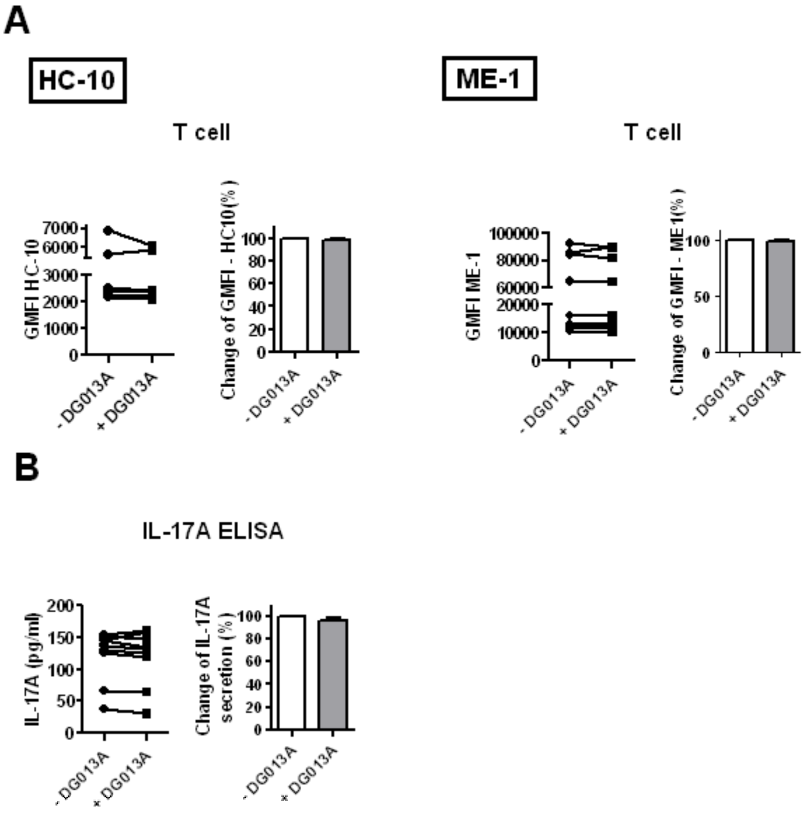

Figure S8 Effect of ERAP1 inhibition on AS patient T cell HLA class I expression and PBMC IL-17A seretion. (A) ERAP1 inhibition by DG013A does not affect HC-10 and ME-1 staining of CD3+ T cells from AS PBMCs. (B) ERAP1 inhibition by DG013A does not affect the IL-17A secretion by AS PBMCs
