# Supplementary material for: Silencing or inhibition of endoplasmic reticulum aminopeptidase 1 (ERAP1) suppresses free heavy chain expression and Th17 responses in ankylosing spondylitis
Source: Ann Rheum Dis. 2015 Jun 30;75(5):916–23. doi: 10.1136/annrheumdis-2014-206996 (PMC4853590; doi:10.1136/annrheumdis-2014-206996)
Supplement: Web methods [file annrheumdis-2014-206996-s2.pdf]

Cell lines

As described previously, HeLa.B27, C1R.B27 and mouse ERAP associated with antigen processing (ERAAP)-/- fibroblasts (ERAAP-/- mFib.B27) were transfected to express HLA- HLA-B*27:05. All three cell lines were cultured in RPMI-1640 supplemented with 10% fetal calf serum, 0.1 mg/ml of streptomycin, and 100 units/ml of penicillin (R10).

Construction and transfection of shRNA-resistant ERAP1 plasmids

To reconstitute ERAP1 in silenced cells, multiple synonymous mutations within shRNA-targeting sequence were delivered to wild type (WT)-ERAP1 plasmid (118-139, from AAA-CGT-AGT-GAT-GGG-ACA-CCA-TTT to AA***G***-CG***A***-AG***C***-GA***C***-GG***A***-AC***G***-CC***T***-TT***C***). The WT-ERAP1 (accession number: BC030775) carries the AS risk alleles of rs30187 and rs27044. The shRNA-resistant WT-ERAP1 plasmid was then further mutated to encode AS-protective ERAP1 variants (K528R, Q730E, K528R/Q730E). All mutations were carried out using quikchange lightning multi site-directed mutagenesis kit (Agilent, USA). These plasmids were transfected into ERAP1-silenced HeLa.B27 or ERAAP-/- mFib.B27 cells using GeneJuice following manufacturer's instructions (Merck Millipore, Germany).

Isolation of peripheral blood mononuclear and $CD4^+$ T cells

Peripheral blood mononuclear cells (PBMCs) were isolated by density gradient centrifugation using Histopaque (Sigma, UK), then frozen and stored in liquid

nitrogen before staining. $CD4^+$ T Cells were negatively isolated from PBMCs using a kit (Miltenyi Biotec, UK). The purity was 90-99% by flow cytometry.

KIR3DL2CD3ε reporter cell assay

Briefly, 200,000 Jurkat T cells transduced with a plasmid expressing the KIR3DL2CD3ε fusion protein were incubated for 2 days with 100,000 HeLa.B27/C1R.B27 in 50 µl/200 µl R10 with/without 50 µg/ml blocking antibodies (HC-10, DX31 and IgG2a). DX31 is a monoclonal IgG2a antibody for KIR3DL2. Supernatants were harvested for IL-2 enzyme-linked immunosorbent assay (ELISA) (EBiosciences).
