# Supplementary material for: Silencing or inhibition of endoplasmic reticulum aminopeptidase 1 (ERAP1) suppresses free heavy chain expression and Th17 responses in ankylosing spondylitis
Source: Ann Rheum Dis. 2015 Jun 30;75(5):916–23. doi: 10.1136/annrheumdis-2014-206996 (PMC4853590; doi:10.1136/annrheumdis-2014-206996)
Supplement: Web table [file annrheumdis-2014-206996-s3.pdf]

**Supplementary Table 1** AS patient demographics

| | **Total** | **rs30187 (T/C)** | | | **rs27044 (G/C)** | | |
|---|---|---|---|---|---|---|---|
| | n=56 | TT n=10 | CT n=26 | CC n=20 | GG n=8 | CG n=22 | CC n=26 |
| **Age, mean ± SD years** | 43.0, ±12.7 | 49.1, ±15.3 | 42.1, ±13.7 | 41.8, ±10.3 | 52.1, ±14.1 | 40.2, ±13.7 | 43.1, ±11.1 |
| **Sex. male/female** | 41/15 | 5/5 | 21/5 | 15/5 | 3/5 | 20/2 | 8/18 |
| **BASDAI, mean ± SD** | 4.3, ± 2.7 | 3.8, ±2.6 | 3.9, ±2.9 | 4.9, ±2.5 | 3.5, ±3.0 | 3.4, ±2.7 | 5.1, ±2.4 |
| **BASFI, mean ± SD** | 3.7, ± 2.8 | 4.1, ±3.5 | 3.6, ±2.6 | 3.9, ±2.9 | 4.1, ±3.5 | 3.0, ±2.4 | 4.3, ±2.9 |
| **BASMI, mean ± SD** | 3.8, ± 2.4 | 2.9, ±2.7 | 4.0, ±2.7 | 3.8, ±2.3 | 2.9, ±2.7 | 3.6, ±2.6 | 4.1, ±2.4 |

AS = Ankylosing Spondylitis; BASDAI = Bath Ankylosing Spondylitis Disease Activity Index; BASFI = Bath Ankylosing Spondylitis Functional Index; BASMI = Bath Ankylosing Spondylitis Metrology Index; none of the patients have been treated with TNF-α blockade (In some cases, score for BASDAI (n=4), BASFI (n=9) and BASMI (n=17) was not available).
